# Geographic and Ethnic Inequalities in Diabetes-Related Amputations

**DOI:** 10.3389/fcdhc.2022.855168

**Published:** 2022-03-11

**Authors:** Alfonso Bellia, Marco Meloni, Aikaterini Andreadi, Luigi Uccioli, Davide Lauro

**Affiliations:** ^1^ Department of Systems Medicine, University of Rome Tor Vergata, Rome, Italy; ^2^ Unit of Endocrinology and Diabetes, University Hospital Policlinico Tor Vergata, Rome, Italy

**Keywords:** lower limb amputation, diabetes, Quality of Life, health care organization and management, geographical disparities

## Abstract

Individuals with diabetes mellitus are at increasing risk for major lower-extremity amputations (LEAs). Poor quality of life and remarkable disabilities are associated with LEAs, determining a high economic burden for the healthcare systems. Reducing LEAs is therefore a primary marker of quality of care of the diabetic foot. At global level, between-countries comparisons of LEAs rates are basically hampered by differences in criteria used for data collection and analysis among studies. Significant variability in amputation rates exists between geographic areas, and also within specific regions of a country. Overall 5-year mortality rate after major amputations is reported to vary substantially across countries, from 50 to 80%. The odds of LEAs are substantially higher for Black, Native American and Hispanic ethnicities compared with White groups, with similar figures observed in the economically disadvantaged areas compared to more developed ones. Such discrepancies may reflect differences in diabetes prevalence as well as in financial resources, health-care system organization and management strategies of patients with diabetic foot ulcers. Looking at the experience of countries with lower rates of hospitalization and LEAs worldwide, a number of initiatives should be introduced to overcome these barriers. These include education and prevention programs for the early detection of diabetic foot at primary care levels, and the multidisciplinary team approach with established expertise in the treatment of the more advanced stage of disease. Such a coordinated system of support for both patients and physicians is highly required to reduce inequalities in the odd of diabetes-related amputations worldwide.

## Introduction

Atherosclerotic vascular diseases and diabetic foot are the conditions that contribute most to the global burden of diabetes mellitus, as measured by disability-adjusted life-years in people aged 50 years and older (1), despite many of these complications being preventable (2). In particular, individuals with diabetes are at increased risk of peripheral arterial disease (PAD) and foot ulcers, which in turn are predisposing factors for major lower-extremity amputations (LEAs) (3). The most important factors predicting poor outcome of foot ulcers are the extent of tissue loss, infections, presence of co-morbidities and PAD (4). However, clinical reasons for a major amputation are quite limited; mostly, LEAs are performed in case of critical limb ischemia with rest pain or progressive infection in a leg that cannot be successfully revascularized. Just in few cases, an immediate amputation is required because of life-threatening sepsis or infection with massive tissue loss. Accordingly, as LEAs are performed in patients with diabetic foot ulcers, they are to be considered as a sort of “final solution” and somehow represent a failure of the previous diagnostic-therapeutic path, that should be based primarily on prevention and multidisciplinary approaches aimed at avoiding - or delaying as much as possible - the use of amputation itself. It follows that high incidence of LEAs can reflect a higher disease prevalence, late referral, limited resources, or a particularly interventionist approach by physicians. Prosthetic limbs are also expensive, often requiring replacement every 3-5 years to account for changes in the body, and demand months of physical rehabilitation therapies for improvement of functional mobility which can be prohibitive for individuals, especially those living in countries economically disadvantaged (5). In addition to loss of mobility and poor quality of life perceived by patients, LEAs result in high economic burden for the healthcare system, since the average life span after a diabetes-related LEA is reported to be roughly five years worldwide (6). For all these reasons, the incidence of LEAs is nowadays considered as a primary marker of the quality of care of the diabetic foot, and reducing diabetes-related LEAs has become a crucial challenge for healthcare providers and the healthcare system (7).

Alarmingly, the number of total amputations has been constantly increasing in the US since the 2007 economic recession, providing an example of how broad economic and racial disparities can lead to disability, reduced quality of life and, eventually, increased mortality (8). These issues have been further magnified by the COVID-19 pandemic, and probably spread to other countries outside the US (9). In fact, patients from disadvantaged groups are often more likely to seek care at safety-net hospitals, which have been overwhelmed with patients in need of admission and intensive care because of COVID-19. These hospitals have been particularly strained during the pandemic and, because of budget constraints, have had less ability to increase their capacity for treating patients with diseases other than COVID-19, when compared with larger metropolitan hospitals.

A number of sociodemographic factors, including ethnicity, financial income, education level and insurance status are responsible for the substantial inequality in diabetes severity observed worldwide, in terms of likelihood of diabetic complications and LEAs (10, 11). In the US both diabetes and LEAs disproportionately affect Black and Hispanic populations, compared with White populations (10–12), and this can be attributed to relevant overlapping social inequalities. Moreover, regional and rural-urban variations in the incidence rates of diabetes and LEAs have been also reported (13). In fact, regional hospital and clinics serving low-income and Black communities may be more likely to perform amputations rather than appropriate evidence-based screening and preventive procedures which could save limbs. According to this background, purpose of this narrative review is to summarize current evidence on existing geographic differences between countries in the rate of diabetes-related LEAs at system level.

## Subsections Relevant for the Subject

A number of determining factors can affect the odds of diabetes-related LEAs and figuring out the reasons why they vary so markedly across different geographic areas and ethnic groups worldwide is challenging. Moreover, between-countries comparisons of diabetes-related LEAs rates are somehow hampered by differences in criteria used for data collection and analysis. In particular, there is paucity of data regarding clinical outcomes determined by diabetes-related LEAs from some parts of the globe, especially from Latin-America, Africa and Oceania, and this represents a major barrier toward the need for increasing awareness of the substantial social, medical, and economic burdens attributable to the diabetic foot syndrome (14–16). Accordingly, overall 5-year mortality rate has been reported to vary substantially, from 29% to 69% following minor amputations, and from 52% to 80% for patients with major amputations (17, 18). Such diversities can be attributable, at least in part, to general disagreement between studies on how to cope with observed variability in results, with different coding mechanisms applied for common definition of diseases and with different methods used for statistical analysis. Notwithstanding, beyond the issue of criteria for collecting data, significant risk factors for mortality following diabetes-related LEAs have been generally recognized the age over 75 years, as well as presence of cardiovascular complications and chronic renal disease (18).

An interesting analysis from Holman et al. (19) had reported significant variations in the recorded incidence of LEAs in England. Based on data reported by all National Health Service (NHS) hospitals over 3 years to March 2010, incidence of total amputations (minor plus major) varied eightfold across Primary Care Trusts in patients with diabetes, ranging from 0.64 to 5.25 per 1,000 person-years. These estimates were in line with previous studies, reporting annual incidence of major amputation in industrialized countries ranging from 0.06 to 3.83 per 10^3^ people at risk (20), and confirmed marked differences in amputation rates occurring between countries and also within specific regions of a country (4). More recently, a large longitudinal analysis on the National Inpatient Sample identified trends in LEAs rates in patients primarily hospitalized with diabetes in the US between 2009 and 2017. The Authors found an increasing annual incidence in LEAs across all racial/ethnic and rural/urban groups, which was primarily driven by increase of minor amputations – by roughly 87% from 2009 to 2017 – whereas major amputations increased by 42% (21). Interestingly, the odds of a major amputation were significantly higher (from 10 to 30% of increase) for Black, Native American and Hispanic ethnicities compared with White groups (21), with similar figures observed in the economically disadvantaged areas compared to more developed ones. These findings are consistent with other recent reports of disproportionately higher rates of LEAs and other diabetes-related complications among racial and ethnic minority populations (10, 22–24). It is unproven that such discrepancies can be attributable to genetic or hereditary factors. Rather, a substantial part of these variations can be explained by differences in disease severity, which can reflect in turn the delay for these minority groups to undergo an early evaluation of diabetic foot ulcerations. Accordingly, in most of these studies lower odds of amputations were significantly associated with having insurance and use of revascularization, confirming how healthcare organization and availability of financial resources have a significant impact on the clinical outcome of diabetic foot ulcers. Additionally, these recent increasing trends in LEAs in the U.S. population came after a period between 1990 and 2010 of substantial decline in non-traumatic amputations and other diabetes-related complications (25, 26), highlighting how the economic recession since 2007 has impacted health system resources and generated inequalities in the access to care for U.S. population.

Detailed global estimates on the disability burden caused by LEAs have been recently provided by an analysis of the Global Burden of Disease (GBD) study (27), obtained from the Institute for Health Metrics and Evaluation (Seattle, Washington) which provided prevalence and years lived with disability (YLDs) estimates for individual diabetes-related lower-extremity complications by 21 regions and 195 countries or territories worldwide. According to 2016 data, an estimated 6.8 million people worldwide received major amputations (with or without prosthesis) consequent to diabetes complications, resulting in 1.6 million YLDs following amputations. These prevalence data of diabetes-related LEAs resulted from an estimated 18.6 million (4.8%) people with diabetes worldwide having a foot ulcer, which is not dissimilar to the 6.3% global pooled prevalence reported in a meta-analysis of 67 eligible studies including 801,985 subjects from 33 countries (28). Of note, prevalence estimates of diabetic foot ulcerations provided by this meta-analysis varied substantially across geographic areas, ranging from 13% for North America to 3% for Oceania, potentially attributable to discrepancies in the screening process for diabetic foot ulcers between countries (29). In the GBD study the overall burden of disease attributable to LEAs appeared to disproportionally affect males, the 50-69 years age group and those living in the regions of North Africa, Central Latin America, Oceania and Middle East (27). Such discrepancies in prevalence estimates of LEAs across geographic areas may reflect, at least in part, differences in diabetes prevalence between countries, since the most affected regions were concomitantly those where diabetes itself is highly prevalent at global level (30). You cannot exclude, however, that inadequacies about health-care systems and financial resources in those geographic territories might be another underlying factors for not performing to levels necessary to cope with the needs of their diabetic population. In accordance, when you compare with 1990 estimates, the 2016 data revealed significant regional changes in age-standardized YLDs attributable to amputations, ranging from a 45.4% increase in Southern Sub-Saharan Africa to an 11.6% decrease in High-Income Asia Pacific (27).

**Figure 1:**
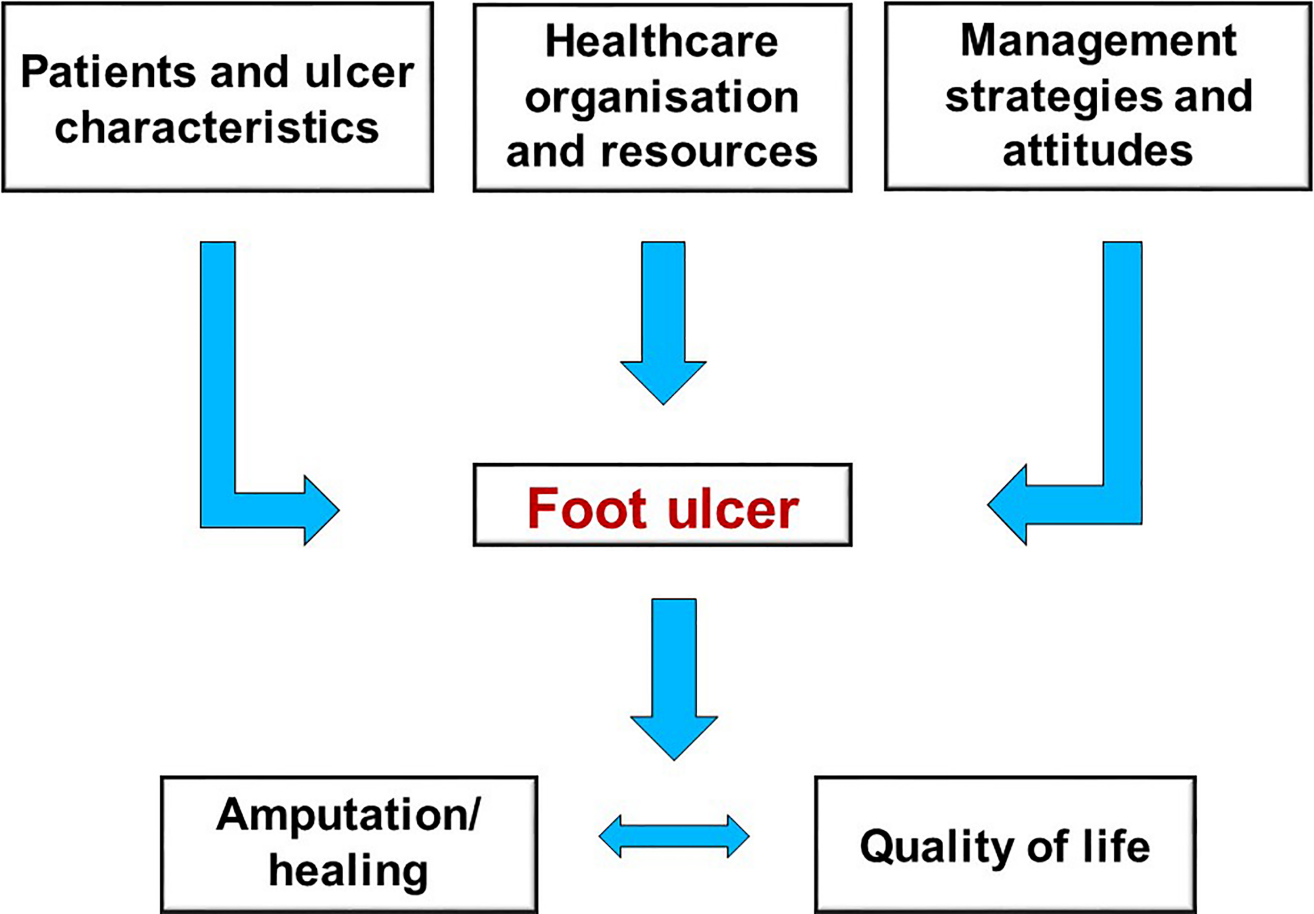
Multiple factors influencing clinical outcome of diabetic foot ulcers.

Further evidence confirming substantial between-countries inequalities in diabetes-related LEAs rates - even among industrialized high-income areas - comes from data collected on 21 countries from different geographic areas by the Organization for Economic Cooperation and Development (OECD) (31). By estimating age– and gender- standardized rates per 100,000 subjects per year between 2000 and 2013, Carinci et al. found mean reduction in major amputations incidence from 182.9 to 128.3 per 100,000 individuals with diabetes (−30.6%) at global levels during the observation period. Noteworthy these incidence estimates, despite the decreasing trend, were dramatically higher when compared with those obtained in the general population (from 10.8 to 7.5 per 100,000 subjects), confirming the remarkable contribution of diabetes per se as risk factor for all the non-traumatic lower limb amputations. Most importantly, Authors reported that age- and sex- standardized rates of diabetes-related LEAs were substantially higher in specific countries, with specific cases of concern being those of Germany (132.2 per 100,000), Israel (158.7 per 100,000), Portugal (188.1 per 100,000) and Slovenia (282.7 per 100,000) (31). By contrast, much lower rates of amputations were found in Italy (49.4 per 100,000) and Luxembourg (48.4 per 100,000). These results can provide further evidence that improving quality of care and clinical outcomes of diabetes foot ulcers, pragmatically evaluable by reduction of LEAs, is not just a problem of financial resources to be specifically allocated to address the issue, but it is also closely related to the specific organization of care and management strategies for diabetic patients adopted by individual countries (**Figure 1**).

## Discussion

Globally, the number of people with diabetes who require insulin, as a measure of complexity of the disease, is estimated at 30-40 million worldwide (https://diabetesatlas.org/). Given to financial disparities between developed and emerging countries in the access to insulin, these patients are the ones who suffer the most from the severe chronic complications that diabetes entails (32). LEAs are among diabetes-related complications primarily affected by social inequalities worldwide (1) and, crucially, the incidence of LEAs is dependent not just on the severity of the disease and the quality of specialist care, but on a number of many confounding medical, social and economic factors. The mortality rate among patients who underwent diabetes-related LEAs is a crucial issue that needs to be addressed through awareness, medical intervention and appropriate legislation (6, 33). The 1989 Saint Vincent declaration - an important initiative set up to address quality and education issues relevant to people with diabetes mellitus in Europe – had already specified the 5-year targets to improve quality of life and life expectancy for people with diabetes mellitus and one of these goals was to reduce the amputation rate by 50% (34). It had also inspired novel research on new approaches for the treatment of diabetic foot and the development of multidisciplinary teams of care. Therefore, it is now essential that diabetes organizations and patient groups may lobby for effective changes of diabetes treatment, in those countries where is more likely to perform amputations and less likely to undertake screening procedures that could save limbs of diabetic patients. In other words, it should be clearly stated that amputation is to be considered as a last resort, after all treatments and therapeutic options have been exhausted.

In this scenario, there is much evidence confirming that a comprehensive evaluation and early intervention can help identify individuals at high risk of diabetic foot and reduce the possibility of hospitalization and LEAs (35, 36). However, one of the obstacles for preventing diabetic foot is the lack of examinations of the feet by primary care practitioners. Accordingly, a 2012 survey from the Institute for Preventive Foot Health/National Purchase Diary survey reported that only 46% of patients with diabetes reported ever having foot screenings with their primary care provider (37). By contrast, regular foot exams on patients with diabetes should be a high priority in primary care setting, and annual comprehensive foot examination to identify risk factors predictive of foot abnormalities and ulcerations are recommended to decrease incidence of LEAs and eventually disabilities and mortality. These foot exams can be easily performed by conventional clinical examination alongside the assessment of sensory neuropathy - through the use of easy tools like the Diabetic Neuropathy Index (DNI) (38) – which are recommended as the screening procedures of choice for foot screening to early detect ulcers and neuropathic foot. Therefore, the first action to reduce the social/economic impact of LEAs should be the implementation of large diabetic foot screening programs at primary care level, to enhance the awareness and relevance of the disease among general practitioners and patients in the early stage of diabetes. The implementation of appropriate exercise therapy programs can be additionally useful in the treatment of patients at risk of diabetic foot. Given the complexity of clinical conditions that patients at risk for diabetic foot ulcer can show, the evaluation of how patients perform the proposed exercise program is consequently of great importance (39). The increased availability of new technologies and in particular of systems and devices equipped with sensors can enable the remote monitoring and management of physical activity performed by patients, particularly in rural and less wealthy areas where telemedicine has been already proposed for neurological diseases and diabetes itself (40). On the other hand, patients with more advanced diabetic foot ulcer should be managed by – and referred to - a multidisciplinary team, with established expertise in revascularization and surgical procedures as well as treatment of infection, oedema, pain, metabolic disturbances, malnutrition, co-morbidities, meticulous wound care and biomechanical offloading (41, 42). A multidisciplinary team oriented to the cure of diabetic foot should be therefore organized with four specific teams: medical, surgical, vascular and rehabilitation teams (42). This complex team approach to diabetic foot has resulted in positive clinical outcomes in terms of reduction of LEAs (43), and more recently has been proposed even for advanced lesion rescue (44). Numerous education and prevention programs have been initiated over the years to combat disparities in diabetes care and outcomes worldwide. Some considerations comes from the Italian experience in the organization model for diabetic foot care. As reported above, lower rates of LEAs were found in Italy (49.4 per 100,000 in 2013) when compared with other countries included in the Organization for Economic Cooperation and Development (OECD) (31). Similarly, an analysis of the National Hospital Discharge Record database in Italy showed a progressive reduction of hospitalization and amputee rates during the period 2000-2010, suggesting an earlier and more diffuse approach aimed at limb salvage (45). These results are likely determined by a number of contributing factors, mostly related to the particular attention given to diabetes - and particularly to the diabetic foot syndrome - by the health care system in Italy. Indeed, the public National Health Service provides free universal coverage and comprehensive healthcare for Italian citizens suffering with diabetes, with the mission to universally provide no-profit high-quality medical care to everyone (46, 47). In addition, a central law issued in 2013, “The Plan on Diabetic Disease”, introduced a multi-centric “reticular” model integrating primary care general practitioners with specialized diabetic teams and giving them the opportunity to share the diagnostic-therapeutic path tailored on the need of the individual patient with diabetes (48). This health care model has generated good results over time in terms of quality of care for patients with diabetes, as demonstrated by decreasing trends in overall mortality (49) and long-term chronic complications (50).

In conclusion, incidence of diabetes-related LEAs can be considered as a measure of quality of care for patients with diabetes, and relevant inequalities across different geographic areas and ethnicity groups unfortunately still exist. Actions should be undertaken to overcome these barriers and to guarantee the delivery of optimal care for the many individuals with diabetic foot disease. It is essential to remark that clinical outcomes of diabetic foot ulcers are not determined just by patient and ulcer characteristics themselves, but also by local healthcare organization, availability of resources, management strategies used and attitudes of the care providers (**Figure 1**). This point has dramatically emerged during the global COVID-19 pandemic, which had a serious and disruptive effect on the delivery of hospital care for those with diabetic foot ulcers. The experience of pandemic has therefore shed light on the necessary reorganization of the care of these fragile patients and on the appropriate application of guidelines. A coordinated system of support for both patients and physicians involved will be highly required to properly address these management strategies in the near future and - by using such an integrated approach – to actually avoid a relevant number of amputations even among disadvantaged populations.

## Author Contributions

AB: Conceptualization, Methodology, Writing—original draft. MM: Conceptualization, Methodology, Writing—original draft. AA: Methodology, Writing—original draft. LU: Writing—review and editing. DL: Supervision. All authors contributed to the article and approved the submitted version.

## Conflict of Interest

The authors declare that the research was conducted in the absence of any commercial or financial relationships that could be construed as a potential conflict of interest.

## Publisher’s Note

All claims expressed in this article are solely those of the authors and do not necessarily represent those of their affiliated organizations, or those of the publisher, the editors and the reviewers. Any product that may be evaluated in this article, or claim that may be made by its manufacturer, is not guaranteed or endorsed by the publisher.
